# Group membership modulates the hold-up problem: an event-related potentials and oscillations study

**DOI:** 10.1093/scan/nsad071

**Published:** 2023-11-22

**Authors:** Su Hao, Qing Xin, Zhang Xiaomin, Pan Jiali, Wang Xiaoqin, Yu Rong, Zhang Cenlin

**Affiliations:** School of Economics and Management, Southwest Petroleum University, Chengdu 610500, China; Key Laboratory of Energy Security and Low-carbon Development, Southwest Petroleum University, Chengdu 610500, China; School of Economics and Management, Southwest Petroleum University, Chengdu 610500, China; School of Economics and Management, Southwest Petroleum University, Chengdu 610500, China; School of Economics and Management, Southwest Petroleum University, Chengdu 610500, China; School of Economics and Management, Southwest Petroleum University, Chengdu 610500, China; School of Economics and Management, Southwest Petroleum University, Chengdu 610500, China; School of Economics and Management, Southwest Petroleum University, Chengdu 610500, China

**Keywords:** hold-up game, group identity, N2, P3, beta band oscillation

## Abstract

This paper investigates the neural mechanism that underlies the effect of group identity on hold-up problems. The behavioral results indicated that the investment rate among members of the in-group was significantly higher than that of the out-group. In comparison to the NoChat treatment, the Chat treatment resulted in significantly lower offers for both in-group and out-group members. The event-related potentials (ERP) results demonstrated the presence of a distinct N2 component in the frontal midline of the brain when investment decisions were made for both in-group and out-group members. During the offer decision-making stage, the P3 peak amplitude was significantly larger when interacting with in-group members compared to the out-group members. The event-related potentials oscillations (ERO) results indicated that when investment decisions were made for in-group members in the NoChat treatment, the beta band (18–28 Hz, 250–350 ms) power was more pronounced than when decisions were made for out-group members. In the NoChat treatment, offer decisions for in-group members yielded a more pronounced difference in beta band (15–20 Hz, 200–300 ms) power when compared to out-group members. Evidence from this study suggests that group identity can reduce the hold-up problem and corroborates the neural basis of group identity.

## Introduction

The theory of the firm delves into the nature, behavior, structure and market relationships of firms, and investigates how they contribute to successful operations both within the economy and internally (Hart, 1995). Moreover, the hold-up problem is a social dilemma that is essential for the theory of the firm, as it is usually found in transactions that necessitate the investment of resources specific to the relationship, when the final distribution of surplus cannot be agreed upon through a contract (Williamson, 1975; Hao *et al.*, 2021a). As the investment is so specific, if bargaining over the surplus fails, the parties have no other options to recoup their investments. When one party takes advantage of the investing party’s lack of an alternative option in the post-agreement negotiations, hold-up can be the result. In such settings, the fear of being held up is likely to dissuade rational agents from making profitable investments, leading to a deadweight loss. Until recently, the impact of group identity on the strategic interaction was largely unexplored. Group identity can serve as a conduit to solve or alleviate the hold-up problem (Morita and Servátka, 2013).

An individual’s self-perception, which derives from social group membership, is referred to as social identity (Turner *et al.*, 1987). Different from social identity, researchers can induce group identity through experimental designs, wherein this group identity supersedes the participants’ intrinsic social identities, such as nationality, ethnicity and gender. Group identity is a concept employed to analyze the behavior of individuals and groups when exposed to a newly established group identity, as well as to evaluate the influences of social identity on economic behavior (Grosskopf and Pearce, 2016). The psychological implications of group membership for the individual and the content of group membership within a particular situation have been theorized to result from social categorization and also to influence subsequent outcomes such as in-group favoritism, identity threat and coping (Tajfel and Turner, 2004). Social categorization is a psychological process in which individuals classify themselves and others into distinct social groups or categories, based on shared characteristics such as gender, race, age and occupation (Hogg and Rinella, 2018). This categorization assists individuals in comprehending complex social circumstances while also influencing their cognition and behaviors. It involves the division of individuals into two distinct groups: in-group members and out-group members, which then leads to a comparison between the two (Rhodes and Baron, 2019). Studies conducted in the early days of psychology have revealed that group membership can result in an in-group bias at the cost of out-group (Tajfel *et al.*, 1971). In-group bias, also known as in-group favoritism, refers to the phenomenon where individuals within a group or society tend to show support, preference and preferential treatment towards members of their own group or in-group (Forbes and Stellar, 2022). Extensive research in the fields of psychology and sociology has found that people are more inclined to establish close connections with members of their in-group and exhibit positive attitudes and behaviors towards them when there are distinctions and recognition between in-groups and out-groups (Su *et al.*, 2023).

In recent years, many economic experiments have studied the interaction between group identity and human decision-making. Charness and Rabin (2002) researched the effect of group identity on social preferences through two-person sequential games, and their results revealed a strong impact of group membership on other-regarding preferences. Through comparing different forms of social categorization, Chen and Li (2009) found that trivial tasks (e.g. painting preferences, t-shirt colors) have the ability to create group effects, although their effectiveness may vary in different environments. However, in comparison, group problem-solving activities such as online chatting significantly enhance attachment to the group. Based on this potential mechanism, Morita and Servátka (2013) proposed that a group identity could be a viable way to address the hold-up problem, and their research found that simply wearing T-shirts as team symbols was not enough to create a powerful enough group identity to resolve the issue. In another study by Morita and Servátka (2016), they randomly assigned participants to two teams and reinforced their group identity through team uniforms and the team online chat task. The results indicated that people within the same team showed higher investment rates and offers in the hold-up game. Investigating group bias in the context of the hold-up problem is critical, as it has already been demonstrated to exist in other economic games. This particular issue arises due to the cooperative relationship and power distribution between the principal and the agent, which includes certain relational investments. Consequently, in-group bias may have a considerable effect on the cooperative relationship, as it involves both parties’ assessment and opinion of the cooperative conditions. Despite the widespread discussion surrounding this topic, to date, no research has been conducted examining the neurological basis of how group identity affects the hold-up problem.

In our event-related potentials (ERP) analyses, we pay attention to N2 and P3 components, which reflect distinct stages of decision-making. The N2, which is a negative component in the frontal central region, has been observed to peak at 200 and 350 ms after the beginning of decision-making. This component is closely related to cognitive control and is particularly sensitive to conflict monitoring and response inhibition. Research has shown that the N2 amplitude generated by high conflict decision-making is larger than that of low conflict decision-making (Nieuwenhuis *et al.*, 2003; Gajewski and Falkenstein, 2013). It is generally accepted that the N2 component is associated with attention allocation (Folstein and Van Petten, 2008; Hao *et al.*, 2023a) and an in-group attention bias in social categorization tasks (Ito and Urland, 2003). The larger N2 component is usually associated with increased attention or cognitive control in cognitively demanding tasks (Folstein and Van Petten, 2008). The N2 component is considered to be an index of attention-related processes, including stimulus discrimination and response selection in the early stages (Olofsson *et al.*, 2008). Evidence suggests that self-bias is contingent upon the availability of attention resources and the anticipation of stimuli (Humphreys and Sui, 2016). The P3 component is a positive waveform, which is most prominent in the central parietal region of the brain, occurring between 300 and 600 ms after the stimulus. The P3 has been demonstrated to have a high sensitivity to the magnitude and valence of reward, displaying more positive amplitude for larger and positive rewards in comparison to smaller and negative rewards (Osinsky *et al.*, 2014). In addition, P3 is thought to reflect the allocation of attention resources, high-level motivation/emotion evaluation (Leng and Zhou, 2010) and psychological ownership (Wang *et al.*, 2021a). Research conducted by Keshvari *et al.* (2019) revealed that P3 amplitude was increased when individuals were presented with both equal and advantageous inequal offers if they were opposed by their in-group members, in regard to group bias on fairness consideration. Corresponding to the group’s norms, in-group members increased their own benefits and provoked a larger P3.

Most studies have only considered the ERP of time-locked and phase-locked responses; yet, event-related oscillations of time-locked but not phase-locked responses may provide further insight into the changes in event-related spectral perturbation (ERSP) energy that are linked to the onset of a stimulus or response, thereby unveiling neural mechanisms related to conflict detection, monitoring and resolution (Tiesinga *et al.*, 2008). Event-related potentials oscillations (ERO) studies on cognitive control have revealed that inhibitory response can lead to an increase in beta band (13 ∼ 30 Hz) energy in the right inferior frontal gyrus, indicating that beta oscillatory can regulate behavior and inhibit potential neural network activities (Alegre *et al.*, 2004; Krämer *et al.*, 2011). Fu *et al.* (2018) found that decisions involving distrust elicited greater beta band energy activity than decisions involving trust. Conversely, Wang *et al.* (2021a) discovered that the trust condition elicited higher frontocentral beta band activity connected to cognitive inhibition than the distrust condition in the trust generation stage.

In sum, this study seeks to explore the effect of created and strengthened group identity on the hold-up problem by analyzing investment rates, offers, ERP (N2 and P3) responses and ERO (beta band) responses. According to related literature (Ito and Urland, 2003; Chen and Li, 2009; Mu and Han, 2010; Morita and Servátka, 2013; Xu *et al.*, 2017; Fu *et al.*, 2018), we hypothesized that, (1) behavioral: investment rates and offers for in-group members would be higher than those for out-group members. Enhanced group identity would increase the investment rates and offers when interacting with in-group members, while reducing it when interacting with out-group members. (2) ERPs: the N2 and P3 amplitudes would be larger for in-group members than for out-group members. Enhanced group identity would produce larger N2 and P3 amplitudes for in-group members than for out-group members. (3) ERSP: the beta band power would be greater for in-group members compared to out-group members. Enhanced group identity would produce a larger beta band power for in-group members than for out-group members.

## Materials and methods

### Participants

We recruited a total of 20 college students, 10 males and 10 females, from the Southwest Petroleum University of China, with ages ranging from 18 to 25 years (M = 20.7 years, SD = 1.89 years) to take part in our experiment. Prior to commencing our study, we performed sample size calculations for conducting a repeated measures analysis of variance using G*Power 3.1.9.7 software (Faul *et al.*, 2007). We selected an effect size parameter of f = 0.25, indicating our expectation of a medium effect size for intergroup differences in our study, based on prior research (Li *et al.*, 2022; Gao *et al.*, 2023) and practical feasibility considerations, including resource and time constraints. The significance level parameter was set at α = 0.05, signifying our willingness to accept a 5% chance of erroneously rejecting the null hypothesis. The statistical power parameter was set at a power of 0.95, indicating a 95% probability of successfully rejecting the null hypothesis when it is false. The final calculation results revealed that a minimum of 13 participants is required to conduct the repeated measures analysis of variance.

There was no history of neurological disorders or mental disease among the participants, who were all right-handed and had a normal or corrected-to-normal vision. Before the experiment, they had to provide written informed consent, which was approved by the Institutional Review Board of the School of Economics and Management at Southwest Petroleum University of China. All participants were given a basic reward of 20 Chinese yuan (roughly US $3.14) and were informed that they would receive extra monetary rewards based on their decisions in the experiment, averaging 42.7 Chinese yuan (approximately $6.7) per participant.

Ten experience sessions were conducted, with four college students instructed to act as interactive participants for two Electroencephalogram (EEG) participants per session. The aim of this recruitment was to guarantee real person interaction and randomness of matching opponents in the experiment. Upon arrival at the laboratory, both EEG participants and interactive participants were informed that the individual in the other room was interacting with them, with the sole distinction that EEG signals were not being recorded from interactive participants.

### Task and procedure

**Fig. 1.**
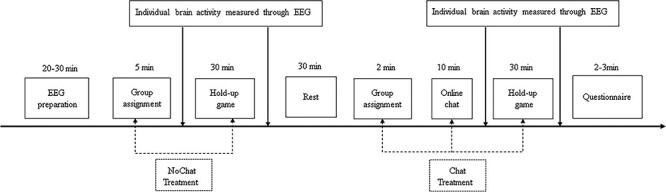
Timeline for an experimental session.

As shown in Figure 1, our experiment was conducted in a within-subject design, with each subject participating in both treatments (NoChat and Chat) based on the induced group identity. Both treatments comprised two tasks: group assignment and playing the repeated hold-up game. Nevertheless, the Chat treatment was distinct in that it included an online chat task before the repeated hold-up game.

Initially, the participants were given instructions to accomplish a group assignment task (Chen and Li, 2009) in order to create group identity. The experimenter handed out five pairs of paintings by two artists to the subjects. In each pair of paintings, one was Klee’s and the other was Kandinsky’s. All subjects independently selected which painting they preferred without knowing each painting’s artist. Consequently, the subjects were divided into two groups: the Klee group and the Kandinsky group, according to their painting preferences. Each group consisted of three individuals, participants were merely informed of the number of individuals in their respective groups, while the precise identities of the members within the group were not disclosed to them. Throughout the experiment, the two groups remained the same throughout experiment.

**Fig. 2.**
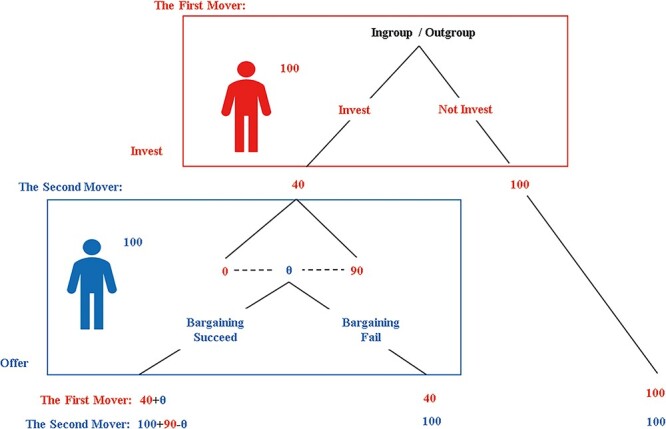
The game tree of the hold-up game.

After the group assignment was completed, the experimenter provided and read aloud the instructions for the hold-up game to all participants. The experiments were conducted with the help of z-Tree software (Fischbacher, 2007) and E-prime software 3.0 (Psychology Software Tools, Sharpsburg, PA, United States). During the experiment, participants’ earnings were substituted with experimental currency units (ECU), each of which was equivalent to 0.004 RMB. Subjects were then randomly paired for a repeated hold-up game between the ‘first mover’ and the ‘second mover’. Before the computer randomly allocated roles to determine earnings, the participants were given an endowment of 100 ECU each and used the strategy method to make decisions as first and second movers at each node of the hold-up game (depicted in Figure 2). This strategy method facilitates the gathering of more observations regarding investment decisions and offers, thus allowing for comparison of behavior across treatments (Hao *et al.*, 2021b). Nevertheless, experiments conducted in the areas of ultimatum bargaining and investment games have not indicated any differences in the outcomes when the strategy method was employed (Brandts and Charness, 2011). Then, the computer randomly allocated roles and tracked all decisions through the ID numbers to calculate the rewards for the participants.

This task had four decision screens, and if the participants were assigned the role of the first mover, they had to decide which strategy to take on the first and second decision screens. We referred to the participant they were paired with as the opponent. The first screen assumed that the opponent was from their own group (in-group condition), while the second screen assumed that the opponent was from the other group (out-group condition). In these two screens, each participant was asked to decide if they would be willing to invest 60 ECU in order to generate a profit of 90 ECU for the team. If they opted not to invest, no other decision was made on the corresponding decision screen. Conversely, if they chose to invest, they had to decide the Minimum Willingness to Accept (MWTA) they were willing to accept in return for the second mover. After the participants had submitted their decisions based on the two scenarios, they proceeded to the third and fourth decision screens. Here, they had to decide the strategy to be taken in case they were assigned the role of the second mover. The third screen assumed that the opponent was from their own group (in-group condition), while the fourth screen assumed that the opponent was from the other group (out-group condition). Each participant determined the offer they would make if their match chose to invest, and after all participants had submitted their decisions under both assumptions, the computer randomly assigned roles within each pair and calculated the earnings of each participant.

The earnings of the hold-up game were determined as follows. If the first mover declined to invest, the respective endowments of the first and second movers were their payoffs. On the other hand, if the first mover chose to invest, two potential results arose. If the second mover’s offer was less than the first mover’s MWTA, the first mover’s payoff was the endowment minus 60 ECU and the second mover’s payoff was the endowment (Bargaining Fail). Conversely, if the second mover’s offer was greater than or equal to the first mover’s MWTA, the first mover’s payoff was the endowment minus 60 ECU plus the second mover’s offer, and the second mover’s payoff was the endowment plus 90 ECU minus the offer (Bargaining Succeed). The outcome screen would provide the participants with the knowledge of whether the person paired with them was an in-group or out-group member.

Once the tasks of the NoChat treatment were finished, the subjects had a 30-minute break during which they were permitted to stand and walk outside; however, they were not allowed to engage in conversation with each other before entering the Chat treatment. After that, the participants were instructed to complete a group chat task through the computer program, based on the group they were assigned to previously. Previous research has demonstrated that online chat enhances group identity in the classic paradigm. In comparison to the Nochat treatment, the Chat treatment exhibits a stronger sense of group identity (Chen and Li, 2009). This task consisted of two questions concerning the other two paintings of the artists, as well as three skill questions (Masella *et al.*, 2014). To help them answer the questions in the online chat task, all participants were provided with five pairs of paintings, each of which had been used in the group assignment task before, and were told the artist corresponding to each painting. Prior to responding to the questions, participants were given a 10-minute period to receive and offer help from their respective groups via online chat. During this time, the Klee group could chat with all its members, and the Kandinsky group could chat with all its members. Once the chat was complete, each participant answered the questions individually. One point was scored for each participant for each correct answer, and the correct answers of each group would be summarized. The group with the highest total score earned an additional 500 ECU for each of its members, whereas the other group did not receive any reward. If the groups are equal in score, the computer would use a random selection process to determine the victor. The purpose of this task was to enhance group identity. All subjects were asked to play the same hold-up game as before after the online chat task was completed. After the experimental tasks were completed, a questionnaire was presented to all subjects that included demographic information such as gender, age and grade.

**Fig. 3.**
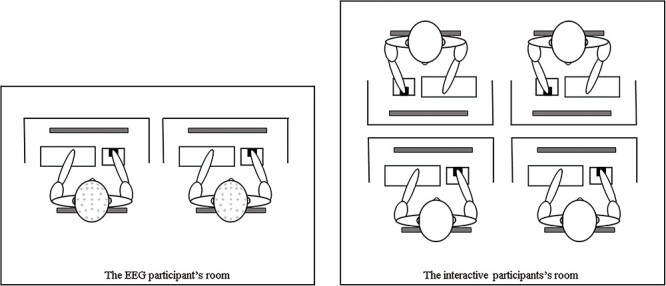
Experimental design sketch. The interactive participants were in a room next to the EEG participant’s room.

**Fig. 4.**
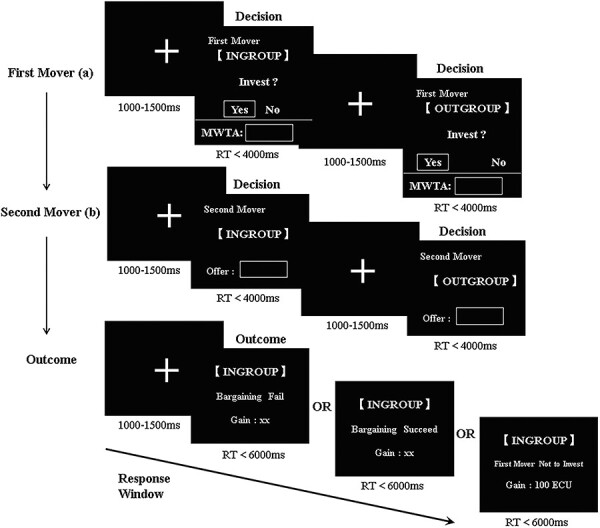
Timeline of a single trial in the hold-up game. This was an example of pairing with people from his own group. In the case of pairing with people from another group, only the display of the outcome screen was different. (a) When participants acted as first movers, they were required to decide whether to make an investment and, if so, input their Minimum Willingness to Accept (MWTA). (b) When participants acted as second movers, they were required to input their offer.

In this case, the two EEG participants and the four interactive participants sat in separate rooms while they completed tasks together using a computer network (shown in Figure 3). The EEG participants were seated comfortably in the electric shielding room, around 70 cm from the computer screen, and then began to don the electrode caps and other preparations. The experimenter introduced the detailed rules of the repeated hold-up game in random pairing to them. An example of an entire trial of the hold-up game can be seen in Figure 4. Each trial started with a fixed cross, which was displayed in the center of the screen for 1000–1500 ms. Participants were then asked to select whether they would invest for an in-group member (if they were first movers) by clicking either the ‘yes’ or ‘no’ button (decision phase), with a maximum time of 4000 ms. Only if they choose to invest would a decision box pop up and it was up to them to decide on the amount of MWTA. The participants were then presented with a fixed cross on the screen for a period of 1000–1500 ms, after which they were prompted to decide whether to invest for an out-group member (if they were first movers) by clicking either the ‘yes’ or ‘no’ button (decision phase), with a maximum time of 4000 ms. Following the presentation of the cross for a duration of 1000–1500 ms, participants were asked to decide the offer they were willing to send for an in-group member (if they were second movers), using the keyboard, for a maximum of 4000 ms (decision phase). A fixed cross was then displayed on the screen for a duration of 1000–1500 ms. Subsequently, participants were asked to make the offer to an out-group member (if they were second movers), using the keyboard, within a maximum of 4000 ms (decision phase). After the cross was visible for a period of 1000–1500 ms, the outcome was then displayed for a maximum of 6000 ms. Then, the computer randomly allocated roles and tracked all decisions through the ID numbers to calculate the rewards for the participants. This outcome would inform the subjects whether the bargaining had been successful and how much they had gained in this trial (outcome phase). Participants had eight practice trials before the formal hold-up game to become acquainted with it. After eight practice trials, participants were eligible to proceed to the formal experiment, which was divided into three sequential parts: the NoChat experiment treatment, the online chat task and the Chat experiment treatment. Both the NoChat and Chat treatments included 50 trials of the hold-up game.

### EEG recording and analysis

Brain electrical activity was measured by the electrode cap of 10–20 system from 64 channels (Neuroscan Inc., USA). All EEG data were recorded with a band-pass filter between 0.05 and 400 Hz, and then were analyzed offline with continuous 1000 Hz sampling. Vertical EOG activity was recorded by electrodes placed above and below the left eye, and horizontal EOG activity was recorded by electrodes at the outer canthi of both eyes. All electrode impedances should remain below 10 kΩ.

The offline EEG data were preprocessed and analyzed by Letswave 7 (Mouraux and Iannetti, 2008), an open-source toolbox running on MATLAB (version R2021a). The EEG data were filtered using Butterworth filters between 0.05 and 35 Hz. Independent component analysis was subsequently implemented to distinguish and remove components associated with eye movements and blinks. EEG epochs were extracted within a time window from −1000 to 2000 ms. After discarding EEG voltages that exceeded ±80 μV, a minimum of 42 trials were retained for each condition. The EEG data were re-referenced using the averaged bilateral mastoids and the baseline was corrected by subtracting the average value of epochs between −200 and 0 ms. The effective trials of each participant were superimposed and averaged, resulting in the generation of ERP datasets of the decision-making stage for the eight conditions.

**Fig. 5.**
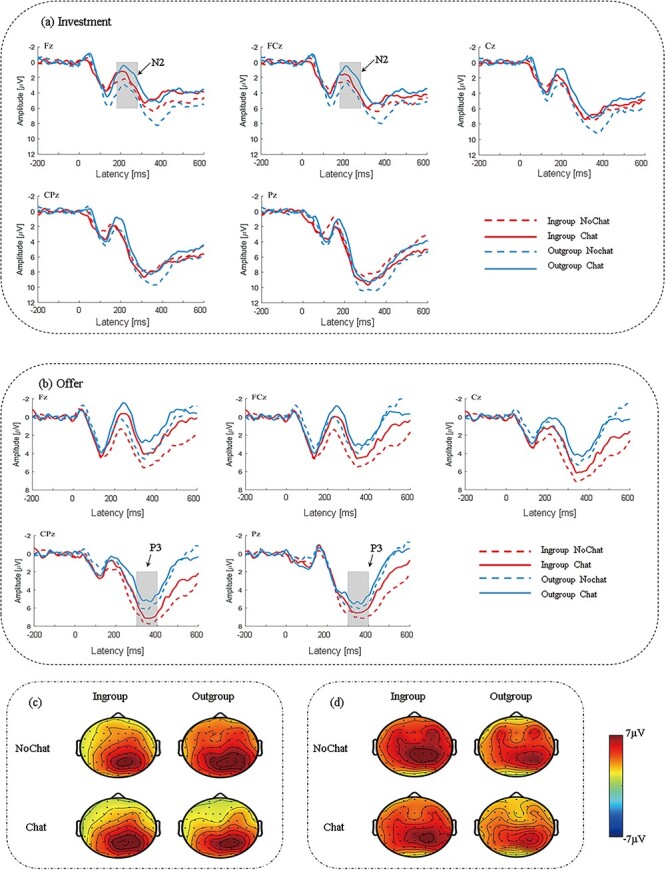
Waveforms and topographic maps. (a) Grand average event-related potentials (ERPs) of investment decision-making at the midline Fz, FCz, Cz, CPz and Pz. The shaded 200–300 ms time window was used for the calculation of the peak amplitudes of the N2. (b) Grand average ERPs of offer decision-making at the midline Fz, FCz, Cz, CPz and Pz. The shaded 300–400 ms time window was used for the calculation of the peak amplitudes of the P3. (c) Topographic maps for the N2 effects in the 200–300 ms time window. (d) Topographic maps for the P3 effects in the 300–400 ms time window.

ERP analyses were conducted from 200 ms prior to the onset of the decision-making interface up to 600 ms after. Based on previous research (Wang *et al.*, 2015; Fu *et al.*, 2018; Wei and Zhou, 2020) and visual observations of the grand average waveforms in the present study (shown in Figure 5), we defined the following components and time windows. In our study, we defined that N2 was the most negative peak in the 200–300 ms time window and P3 was the most positive peak in the 300–400 ms time window. The N2 and P3 were employed to analyze the investment and offer decision-making processes, respectively. According to the scalp distribution and previous literature (Wang, 2010; Wang *et al.*, 2015; Fu *et al.*, 2018), we selected five midline electrode sites (Fz, FCz, Cz, CPz and Pz) for the N2 and P3 analyses. Statistical analysis was conducted by SPSS software version 24 (SPSS Incorporation, Chicago, IL, United States). A three-factor repeated measures ANOVA of 2 (group membership: in-group vs. out-group) × 2 (online chat: no vs. yes) × 5 (electrodes: Fz, FCz, Cz, CPz, Pz) was conducted for the most wave amplitude. After using the Greenhouse-Geisser method to correct the *P*-values of main and interaction effects for repeated measurement effects, only significant effects were reported. Additionally, the Bonferroni correction was employed for multiple comparisons.

### Time-frequency analysis

After the reprocessing of all EEG data, Letswave software was employed to carry out continuous Morlet wavelet transforms (CWT) in order to acquire oscillation power estimations on time and frequency from single-trial EEG epochs. A time window from −1000 to 2000 ms was used to extract single-trial epochs relative to stimulus onset, and a 500 Hz down-sampling of EEG data was performed. The mother wavelet was set to cmor1 ∼ 1.5, allowing for the exploration of time-frequency representations between 3 Hz and 35 Hz (Wang *et al.*, 2017; Fu *et al.*, 2019). In order to identify the modulations of ongoing ERSP, time-frequency representations of each trial were averaged to obtain averaged time-frequency representation. For each estimated frequency, ERSP magnitudes were displayed as an increase or decrease in oscillatory power relative to the pre-stimulus interval (−400 to −200 ms) according to the following formula: $ER_{t,\;f}\%=\frac{A_{t,\;f}-R_f}{R_f}$, where $A_{t,\;f}$was the signal power at a given time (t) and frequency (f), and $R_f$ was the signal power averaged within the pre-stimulus interval (Pfurtscheller and Da Silva, 1999). The pre-stimulus time interval (−400 to −200 ms) was used as a baseline interval to prevent edge effects during CWT.

**Fig. 6.**
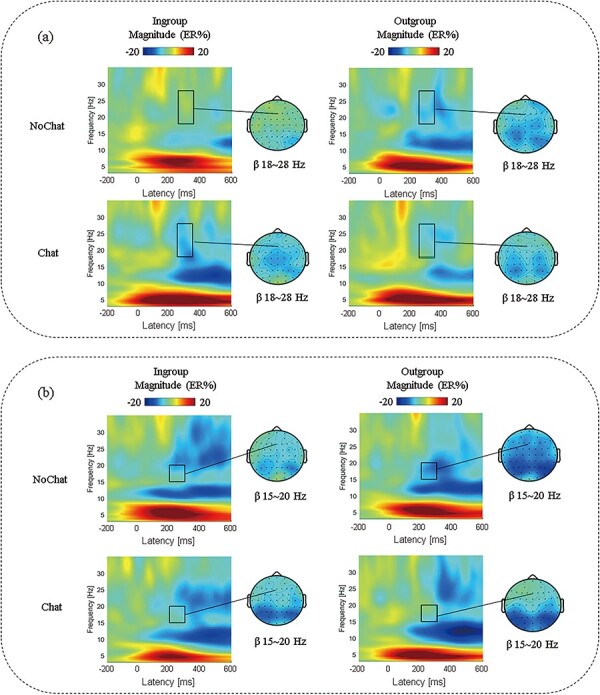
The grand-average time-frequency results at Fz. Dark rectangles mark the time-frequency window used in the statistical analysis and the corresponding scalp topographies. (a) The grand-average time-frequency representations (expressed as ER %, 250–350 ms) for each investment decision-making condition. (b) The grand-average time-frequency representations (expressed as ER %, 200–300 ms) for each offer decision-making condition.

Then, consistent with previous research (Marco-Pallares *et al.*, 2008; Krämer *et al.*, 2011; Wang *et al.*, 2021a), and based on visual inspection of group-averaged time-frequency spectrograms (shown in Figure 6), we calculated the mean magnitude of beta band activities across the frequency of 15–20 Hz within a 200–300 ms time window and the frequency of 18–28 Hz within a 250–350 ms time window after stimulus onset. Statistical analyses were performed on midline electrode sites Fz, FCz, Cz, CPz and Pz. A three-factor repeated measures ANOVA of 2 (group membership: in-group vs. out-group) × 2 (online chat: no vs. yes) × 5 (electrode: Fz, FCz, Cz, CPz, Pz) was conducted for the beta band power. After using the Greenhouse-Geisser method to correct the *P*-values of main and interaction effects for repeated measurement effects, only significant effects were reported. Additionally, the Bonferroni correction was employed for multiple comparisons.

## Results

### Behavioral results

Table 1 shows a summary of the statistics from the two treatments. In Table 1, the *P*-value of each test statistic is presented in the bottom portion when distinguishing between two treatments, and in the rightmost column when evaluating differences between groups. Taking into account the non-normality of behavioral data, a paired samples Wilcoxon signed rank test was conducted which showed that the investment rate in the in-group condition was significantly higher than the out-group condition in both the NoChat treatment (*P* < 0.001) and Chat treatment (*P* = 0.014). However, there was no significant difference in investment rate between the NoChat treatment (mean ± SD, 0.913 ± 0.184) and the Chat treatment (mean ± SD, 0.881 ± 0.218) under in-group condition (*P* = 0.135), or between the NoChat treatment (mean ± SD, 0.635 ± 0.407) and the Chat treatment (mean ± SD, 0.584 ± 0.409) under out-group condition (*P* =0.414).

**Table 1.** Summary statistics

		*In-group*	*Out-group*	*H_0_:In-group = Out-group*
*NoChat* *treatment*	Invest	91.3%	63.5%	*P-value* = 0.0004
	Offer	57.53 ECU	53.39 ECU	*P-value* = 0.3042
*Chat* *treatment*	Invest	88.1%	58.4%	*P-value* = 0.0143
	Offer	53.26 ECU	44.03 ECU	*P-value* = 0.1556
*H_0_:NCT = CT*	Invest	*P-value* = 0.1346	*P-value* = 0.4143	
	Offer	*P-value* = 0.0317	*P-value* = 0.0019	

Notes: The *P*-values are derived from paired samples Wilcoxon signed rank test.

Then, a paired samples Wilcoxon signed rank test showed that the offer in the NoChat treatment was significantly higher than that in the Chat treatment for both in-group (*P* = 0.032) and out-group (*P* = 0.002) conditions. However, there was no significant difference of offer between the in-group (mean ± SD, 57.53 ± 17.387 ECU) and out-group (mean ± SD, 53.39 ± 20.950 ECU) conditions in the NoChat treatment (*P* = 0.304), or between the in-group (mean ± SD, 53.26 ± 21.973 ECU) and out-group (mean ± SD, 44.03 ± 25.658 ECU) conditions in the Chat treatment (*P* = 0.156).

Ultimately, the results of the paired samples Wilcoxon signed rank test indicated that the MWTA for the NoChat treatment was significantly greater than the MWTA for the Chat treatment in both in-group (*P* < 0.001) and out-group (*P* < 0.001) conditions. Moreover, there was a significant difference in the MWTA between the in-group (mean ± SD, 48.57 ± 21.924 ECU) and out-group (mean ± SD, 34.39 ± 30.130 ECU) conditions in the NoChat treatment (*P* < 0.001), as well as between the in-group (mean ± SD, 44.20 ± 24.117 ECU) and out-group (mean ± SD, 28.87 ± 29.659 ECU) conditions in the Chat treatment (*P* < 0.001). The behavioral results showed that the in-group had a greater rate of investment compared to the out-group, and the Chat condition had a substantially diminished offer and the MWTA in comparison to the NoChat condition. Additionally, the MWTA in the NoChat/Chat conditions was higher for the in-group than for the out-group.

### ERP results

Figure 5 shows the grand-average ERP waveforms and topographic maps of 20 participants. We selected the N2 and P3 components of the grand-average waveforms for further statistical analyses. Then, a repeated measures ANOVA of 2 (group membership) × 2 (online chat) × 5 (electrode) was performed on each component. In Figure 5, we only focused on the N2 component but neglected the P3 component in the ‘Investment’ phase. On the contrary, we analyzed the P3 component but neglected the N2 component in the ‘Offer’ phase. This was because the statistical results of the P3 component in the ‘Investment’ stage and the N2 component in the ‘Offer’ stage were not significant.

#### N2

The main effect of electrode was significant, F (4, 152) = 24.547, *P* < 0.001, η^2^ = 0.392, suggesting that the N2 peak amplitude increased from front to back. The N2 peak amplitude in the frontal midline (Fz) was the smallest (mean ± SE, 0.409 ± 0.569 µV), and in the parietal midline (Pz) was the largest (mean ± SE, 3.526 ± 0.604 µV). The interaction between group membership and electrode was significant, F (4, 152) = 4.39, *P* = 0.02, η^2^ = 0.104. Further simple effects found that the peak amplitudes of the five electrodes were significantly different under in-group and out-group conditions, respectively. Post-hoc analysis indicated that the N2 peak amplitude at Fz (mean ± SE, 0.482 ± 0.592 µV) was significantly smaller than that of other electrodes (Cz: mean ± SE, 1.9 ± 0.642 µV, *P* = 0.001; CPz: mean ± SE, 2.653 ± 0.623 µV, *P* < 0.001; Pz: mean ± SE, 4.075 ± 0.607 µV, *P* < 0.001) under the in-group condition. Under the out-group condition, the N2 peak amplitude at Fz (mean ± SE, 0.336 ± 0.697 µV) was only significantly smaller than that of Pz (mean ± SE, 2.977 ± 0.773 µV) (*P* = 0.001), but not significantly different from Cz (mean ± SE, 1.163 ± 0.736 µV, *P* = 0.127) and CPz (mean ± SE, 1.436 ± 0.731 µV, *P* = 0.071). The main effect of online chat was not significant, F (1, 38) = 1.067, *P* = 0.308, η^2^ = 0.027. Overall, during the ‘investment’ phase, the N2 peak amplitudes of all five electrodes were significantly higher under the in-group condition compared to the out-group condition. Additionally, all five electrodes were examined under the in-group condition, and the N2 peak amplitude at Fz was the smallest of the group.

#### P3

The main effect of group membership was significant, F (1, 38) = 9.497, *P* = 0.004, η^2^ = 0.20. When the opponent was assumed to be an in-group member, the induced P3 peak amplitude (mean ± SE, 7.156 ± 0.582 µV) was significantly larger than that of out-group members (mean ± SE, 5.714 ± 0.572 µV). The main effect of electrode was significant, F (4, 152) = 16.459, *P* < 0.001, η^2^ = 0.302, indicating that the P3 peak amplitude increased from front to back. The peak amplitude in the frontal midline (Fz) was the smallest (mean ± SE, 5.160 ± 0.609 µV), and in the central parietal midline (CPz) was the largest (mean ± SE, 7.620 ± 0.541 µV). The interaction between group membership and electrode was significant, F (4, 152) = 4.644, *P* = 0.011, η^2^ = 0.109. Further simple effects found that when the in-group and out-group conditions were compared directly over five electrodes, group membership did affect P3 peak amplitude at five electrodes. Post-hoc analysis indicated that the induced P3 peak amplitude under in-group condition (mean ± SE, 5.746 ± 0.686 µV) in the frontal midline (Fz) was significantly larger than that induced under out-group condition (mean ± SE, 4.574 ± 0.637 µV) (*P* = 0.03). The induced P3 peak amplitude under in-group condition (mean ± SE, 5.982 ± 0.676 µV) in the frontal central midline (FCz) was significantly larger than that induced under out-group condition (mean ± SE, 4.559 ± 0.681 µV) (*P* = 0.015). The induced P3 peak amplitude under in-group condition (mean ± SE, 7.548 ± 0.621 µV) in the central midline (Cz) was significantly larger than that induced under out-group condition (mean ± SE, 5.676 ± 0.644 µV) (*P* = 0.001). The induced P3 peak amplitude under in-group condition (mean ± SE, 8.519 ± 0.587 µV) in the frontal central parietal midline (CPz) was significantly larger than that induced under out-group condition (mean ± SE, 6.720 ± 0.598 µV) (*P* = 0.001). The induced P3 peak amplitude under in-group condition (mean ± SE, 7.986 ± 0.639 µV) in the frontal-parietal midline (Pz) was significantly larger than that induced under out-group condition (mean ± SE, 7.041 ± 0.604 µV) (*P* = 0.017). The main effect of online chat was not significant, F (1, 38) = 1.095, *P* = 0.302, η^2^ = 0.028. In summary, the group membership significantly influenced the induced P3 peak amplitude, with higher amplitudes observed when the opponent was assumed to be an in-group member.

### ERSP results

The grand-average time-frequency graphs of 20 participants at electrode Fz are shown in Figure 6. A repeated measures ANOVA of 2 (group membership) × 2 (online chat) × 5 (electrode) was performed on the mean amplitudes of beta power.

#### ERSP results for investment decision-making

The ANOVAs of the beta-band (18–28 Hz, 250–350 ms) revealed a significant interaction of group membership × online chat × electrode, F (4, 152) = 3.517, *P* = 0.036, η^2^= 0.085. A further simple test showed that the beta power had different responses to different treatments under the in-group condition at electrode Fz, F (1, 38)= 6.132, *P* = 0.018, η^2^= 0.139, with NoChat treatment (mean ± SE, −0.1 ± 1.8 ER%) eliciting larger beta power than Chat treatment (mean ± SE, −6.6 ± 1.8 ER%). In contrast, two treatments in the out-group condition did not significantly modulate beta power over five electrodes, all *P* > 0.489. A direct comparison of in-group and out-group conditions for each treatment over five electrodes showed that beta power was affected by group membership in the NoChat treatment at electrode Fz, F (1, 38)= 8.916, *P* = 0.005, FCz, F (1, 38)= 9.088, *P* = 0.005, Cz, F (1, 38)= 9.190, *P* = 0.004 and CPz, F (1, 38)= 6.816, *P* = 0.013. At these four electrodes, beta power was larger under the in-group condition (mean ± SE, Fz: −0.1 ± 1.8, FCz: −0.6 ± 2.0, Cz: −1.7 ± 2.1, CPz: −2.0 ± 2.0 ER%) than under out-group condition (mean ± SE, Fz: −5.5 ± 1.8, FCz: −6.6 ± 1.8, Cz: −7.5 ± 2.0, CPz: −7.9 ± 2.1 ER%). Based on the findings, no significant variance was noted in beta power between the NoChat and Chat treatments under the out-group condition. However, when comparing the in-group and out-group conditions within the NoChat and Chat treatments, all five electrodes revealed a significantly higher beta power under the in-group condition in comparison to the out-group condition.

#### ERSP results for offer decision-making

The ANOVAs of the beta-band (15–20 Hz, 200–300 ms) revealed a significant main effect of electrode, F (4, 152) = 7.041, *P* = 0.002, η^2^ = 0.156, suggesting that the beta power decreased from the front to back. The beta power in the frontal midline (Fz) was the largest (mean ± SE, −5.2 ± 1.6 ER%), and in the parietal midline (Pz) was the lowest (mean ± SE, −9.3 ± 2.0 ER%). The interaction between group membership and online chat was marginally significant, F (1, 38) = 3.872, *P* = 0.056, η^2^ = 0.092. A further simple test found that the beta power under in-group and out-group conditions was significantly different in the NoChat treatment. Post-hoc analysis indicated that in the NoChat treatment, the beta power of the in-group condition (mean ± SE, −5.8 ± 2.9 ER%) was significantly larger than that of the out-group condition (mean ± SE, −10.1 ± 2.5 ER%) (*P* = 0.029). To summarize, the frontal midline (Fz) had the highest beta power observed, with the parietal midline (Pz) having the lowest. Additionally, the beta power in the NoChat treatment showed significant differences between in-group and out-group conditions.

## Discussion

While there is a wealth of literature that has looked into the hold-up game, no research has yet employed EEG technology to investigate this topic. Our research focused on the neurophysiological correlates of group identity and how they were reflected in the participants’ decisions in the hold-up game. Utilizing ERP and ERO technology, we examined the dynamic temporal characteristics of brain activity in the decision-making stage of creating and strengthening group identity. The behavioral results revealed that the investment rate of in-group members was significantly higher than that of out-group members. The offers and MWTA for in-group and out-group members in the Chat treatment, which strengthened group identity through an online group chat, were significantly lower than those in the NoChat treatment, which only created group identity through categorization. Additionally, the MWTA of the in-group was higher than that of the out-group in both the NoChat and Chat conditions. The ERP results found that there was an obvious N2 component in the frontal midline of the brain when making investment decisions for both in-group and out-group members, indicating that the brain might be sensitive to group identity. The P3 peak amplitude was significantly higher when interacting with in-group members compared to out-group members. Moreover, on the midline of the brain, the P3 peak amplitude for offers decision-making for in-group members was significantly larger than that for out-group members. The ERSP results showed that the difference of the beta band (18–28 Hz, 250 ∼ 350 ms) power was more pronounced when investment decisions were made for in-group compared to out-group members in the NoChat treatment. Furthermore, the beta band was greater when the group identity was created only by categorization than when it was augmented by categorization and online chat. In the NoChat treatment, the beta band (15–20 Hz, 200 ∼ 300 ms) power was more pronounced when offer decisions were made for in-group members as opposed to out-group members. These findings demonstrated that the group identity could alleviate the hold-up problem and further affirmed the neural basis of group identity.

Firstly, the behavioral results indicated that group identity had a positive effect in alleviating the hold-up problem. The investment rate of in-group members was significantly higher than that of out-group members, indicating that group membership mitigated the inefficiency associated with investments in specific relationships (Morita and Servátka, 2013). Although the offers for in-group members were higher than those for out-group members, the disparity was not statistically significant. In the Chat treatment, the offers for both in-group and out-group members were significantly lower than those in the NoChat treatment, which was not in line with our prediction. This outcome could be attributed to the strategic element of the hold-up game, which appears to have eliminated any in-group favoritism, and thus, in-group members were not granted preferential treatment unconditionally (Yamagishi and Kiyonari, 2000). During the NoChat and Chat treatments, the MWTA for members of the in-group was significantly greater than for those of the out-group. This phenomenon may be due to an increase in in-group favoritism, leading participants to increase their investment rates in in-group members and expect equal returns in interactions, known as reciprocity (Gouldner, 1960; Hao *et al.*, 2023b). Additionally, participants believe that within the same group, there should be a mutually skewed reciprocal relationship among members, rather than unilateral giving (Gervasi *et al.*, 2022). Consequently, participants’ MWTA from in-group members is significantly higher than from out-group members.

Secondly, our ERP results revealed that the N2 and P3 components were modulated by group identity. Consistent with previous studies (Wang *et al.*, 2015), we observed a distinct N2 component in the frontal midline of the brain during investment decision-making for both in-group and out-group members. Moreover, the results indicated that the N2 peak amplitudes from all five electrodes were significantly enhanced under the in-group condition as compared to the out-group condition. This finding can be potentially explained in the following ways: From one perspective, it can be interpreted as enhanced in-group favoritism, where participants experienced increased in-group preference either before or after the online chat task. Previous research suggests that the amplitude of the N2 component tends to increase when individuals engage in in-group categorization (Dickter and Bartholow, 2007). This aligns with our findings that the N2 amplitude was higher when participants interacted with in-group members, indicating a preferential focus on in-group cues (Ito and Urland, 2003). Additionally, according to Derks *et al.* (2015), the N2 component was significantly impacted by social identity. From another perspective, another possible explanation relates to cognitive control and attentional processes. It is generally acknowledged that the N2 component is linked to attention allocation (Folstein and Van Petten, 2008) and an in-group attention bias in social categorization activities. Furthermore, a greater N2 component is usually associated with augmented attention or cognitive control in cognitively demanding tasks (Ito and Urland, 2003). In addition, existing evidence suggests that self-bias depends on the availability of attentional resources and the anticipation of stimuli (Humphreys and Sui, 2016). Some scholars argue that due to the influence of social identity on the N2 component, it implies that social identification and threats to social identity do not determine which groups are perceived as threatening, but rather which targets receive more attentional resources (Derks *et al.*, 2015). In terms of cognitive control, the N2 component is involved in regulating cognitive processes and is particularly sensitive to monitoring conflicts and inhibiting responses (Nieuwenhuis *et al.*, 2003; Gajewski and Falkenstein, 2013).

In addition, results revealed that P3 amplitude was significantly higher when interacting with in-group members compared to out-group members, thus demonstrating the influence of group membership on P3 amplitude. The P3 is considered to reflect motivational salience, attention allocation and high-level affective/social evaluation (Leng and Zhou, 2010). It has been suggested that a larger P3 amplitude may be the result of increased attention resources and stronger emotional responses to psychologically owned items (Johnson *et al.*, 2002). A study conducted by Keshvari *et al.* (2019) found that P3 amplitude increased when people were exposed to equal and advantageous inequal offers from those in their in-group, indicating the influence of group bias on fairness consideration. Moreover, P3 was believed to be a neural representation of psychological ownership (Wang *et al.*, 2021a). Turk *et al.* (2011) found that self-ownership cues were associated with increased attention processing, measured by the P3 component, even if the basis of ownership was arbitrary and transient. An enhanced P3 amplitude indicated that more early attention resources were used to distinguish the highly important self-related content from minimally important content. When subjects evaluated an object as self-related, the index of attention processing would increase (Xu *et al.*, 2017). Self-relevant and in-group-relevant cues led to us perceiving our own objects as more attractive, valuable and having a higher memory advantage (De Bortoli Vizioli *et al.*, 2020). Our findings were in agreement with the notion that the P3 amplitude generated by in-group members was higher than that generated by out-group members, as participants had a greater sense of ‘extended self’ towards in-group members. A large number of studies have demonstrated that P3 is an important indicator of prosocial motivation, with a higher P3 amplitude indicating a heightened prosocial motivation (Carlson *et al.*, 2016). However, there was no significant difference in P3 amplitudes between the NoChat and Chat treatment. The absence of a rise in subjects’ self-related cognition might be attributed to the fact that the group identity that was strengthened through online chat alone was insufficient.

Finally, our ERSP results indicated that beta band power was also modulated by group membership. Beta band oscillations have been identified as a key factor in the projection of top-down processes, which can be observed in the inter-areal coherence related to selective attention (Saalmann *et al.*, 2007), working memory (Tallon-Baudry *et al.*, 2001), guided search (Buschman and Miller, 2009) and object recognition (Sehatpour *et al.*, 2008). Notably, this coherence was particularly evident in the beta range. The beta reduction decreases in inhibited trials corroborate previous findings of beta power changes associated with response inhibition in the Stop-Signal task (Marco-Pallares *et al.*, 2008;) and Go/No-Go tasks (Alegre *et al.*, 2004). The difference in beta band power induced by different decision types showed that distrust selection involved stronger cognitive control processing than trust selection, which was not the dominant option that consumed more cognitive resources (Fu *et al.*, 2018). However, few EEG studies have been conducted on self-reference processes. Mu and Han (2010) observed event-related desynchronization (ERD) related to self-referential traits in beta band activity over the central-parietal area through time-frequency analyses. Our study revealed that there was a greater beta band power in two stages of decision-making when participants interacted with in-group members as opposed to out-group members. Our findings implied that beta oscillations might (at least partially) be implicated in the N2 and P3 effects observed in the EEG time domain. In addition, we observed no considerable variation in beta band energy between group identity created only by categorization and group identity reinforced by categorization and online chat. This could be attributed to the inadequate enhancement of group identity through online chat to significantly modify participants’ cognitive control.

It is essential to admit that this study is subject to certain restrictions and deficiencies. Initially, our research divided participants based on their art preferences, and in the future, we plan to randomly assign individuals to the groups to evaluate the level of emotional engagement and its impact on the group’s identity. Furthermore, due to the adoption of a within-subject design, the confounding of order effects and learning effects is inevitable. Despite our pre-experiment procedure to try to mitigate this issue, we cannot deny its existence. Thirdly, this study was unable to provide key information regarding the response time (RT) of participants under different conditions, as the accuracy of the z-Tree software was limited to seconds. It is hoped that future studies will explore if any distinctions in RT exist between participants in different conditions, and the findings should be reported. Moreover, given the limitations of the experimental technology, we were unable to acquire the MWTA and the influence of this decision on the results. Ultimately, our goal in future research is to utilize machine learning algorithms, such as Support Vector Machines (SVM) and Random Forests, to train on extracted features from EEG data and predict the investment outcomes of participants in the hold-up game. By analyzing the predictive results, we can uncover the associations between distinct neural patterns and investment decisions.

## Conclusion

Our study indicated that group membership had an effect on the attention allocation and cognitive control in the hold-up game, which was observed over different time courses. The ERP study revealed that interacting with in-group members caused a rise in the amplitude of the N2 component, suggesting that attention was biased towards in-group cues. And the P3 amplitude generated by in-group members was larger than that generated by out-group members, as more early attention resources were devoted to distinguishing the highly important self-related content from minimally important content. The increased allocation of attention resources to in-group members might lead to a larger P3 amplitude. Additionally, beta oscillation might (at least partially) be the basis of N2 and P3 effects in the EEG time domain, thus demonstrating group identity characteristics.
